# Association between Socioeconomic Status and Vaccination Hesitancy, Reluctancy and Confidence among Asian-Americans Living in the State of New Jersey

**DOI:** 10.1007/s10900-024-01381-2

**Published:** 2024-07-16

**Authors:** Brijesh Rana, Humberto R. Jimenez, Zeba M. Khan, Navaneeth Narayanan

**Affiliations:** 1 School of Public Health, Rutgers, The State University of New Jersey, 683 Hoes Lane West, Piscataway, NJ 08854 USA; 2 Rutgers Cancer Institute of New Jersey, 195 Little Albany Street, New Brunswick, NJ 08901 USA; 3 Rutgers University Ernest Mario School of Pharmacy, 160 Frelinghuysen Rd, Piscataway, NJ 08854 USA

**Keywords:** Asian-Americans, SES, Vaccine Hesitancy and Confidence

## Abstract

**Backgroud:**

Socioeconomic status (SES) plays a vital role in determining vaccination uptake and attitudes. Vaccine hesitancy varies among different communities, yet knowledge of vaccine attitudes among Asian-Americans is limited.

**Objective:**

This study aims to investigate the relationship between SES and vaccine attitudes among Asian-Americans in the State of New Jersey (NJ).

**Methods:**

Asian-Americans aged ≥ 18 years living in NJ were included (*N* = 157). SES was measured by education level, employment type, employment status, and household income. The primary outcomes were vaccine hesitancy, reluctance, and confidence for COVID-19, influenza, and pneumococcal vaccines. Descriptive and inferential statistics were performed. Multivariable logistic regression was used to identify associations between SES and vaccine hesitancy while controlling for confounders such as age, gender, birthplace, and religion.

**Results:**

Among 157 participants, 12.1% reported vaccine hesitancy. There was no statistically significant association between vaccine hesitancy and education level (*p* = 0.68), employment status (*p* = 1), employment type (*p* = 0.48), and household income (*p* = 0.15). Multivariable logistic regression modeling confirmed that none of the SES predictor variables were associated with vaccine hesitancy. However, as exploratory finding, gender was found to be a significant predictor, with males having lower odds of vaccine hesitancy than females (Adjusted OR = 0.14; *p* < 0.05). Confidence in influenza and pneumococcal vaccines increased during the pandemic, from 62.34% to 70.13% and from 59.2% to 70.51%, respectively. For the COVID-19 vaccine, 73.1% of participants reported having “a lot of confidence” in taking vaccine.

**Conclusion:**

Most sampled Asian-Americans in NJ have high confidence in taking COVID-19 vaccines, and there is no significant association between vaccine hesitancy and SES.

## Introduction

Socioeconomic status (SES) has been recognized by public health specialists as one of the most significant determinants of preventative health behavior since the last 50 years [1]. Despite considerable evidence supporting the safety of vaccines, skepticism regarding vaccination continues to grow [2]. Individuals of different cultural backgrounds have diverse perceptions of vaccine risk, and there is existing research demonstrating a relationship between SES and vaccine reluctancy [2]. SES is an important component in vaccine hesitation, trust, and willingness [3]. The COVID-19 pandemic has disproportionately affected low SES groups, with the higher rates of illness and mortality, as well as significant economic impacts due to public health measures such as lockdowns [4].

National immunization programs and the World Health Organization’s (WHO) Strategic Advisory Group of Experts (SAGE) on Immunization have emphasized the need for improved monitoring of vaccine-related behaviors to inform the creation of communication and other interventions to address confidence gaps, to maintain trust in vaccines and immunization programs [5]. Moreover, pneumococcal and influenza vaccinations were strongly advised during the COVID-19 pandemic to protect the most marginalized individuals and groups [6]. Influenza and pneumococcal vaccination rates among Asian Americans are not well described due to disproportionately low representation in population survey studies. One study described Asian American immunization rates similar to those of white Americans [7]. Particularly, South Asians are one of the racial groups in New Jersey that is growing the quickest, and the state has the third-largest population of South Asians in the US [8]. Hence, it becomes essential to learn about the vaccine related behaviors. Through this study, the primary aim is to understand COVID-19, influenza and pneumococcal vaccine related behaviors among Asian-Americans living in New Jersey and the impact of SES (education, household income and employment status) affecting these behaviors.

## Methods

### Study Design and Population

The proposed study is an observational study that utilizes data from survey records to examine the association between socioeconomic status (SES) (education, employment, and household income) and vaccination attitudes for influenza, pneumococcal, and COVID-19 vaccines among Asian-Americans living in New Jersey. The primary survey was distributed between June 2022 and April 2023 at 8 diverse clinical or community sites across New Jersey. These sites included Center for Comprehensive Care in Jersey City - Jersey City Medical Center at Greenville, Department of Community Medicine at St. Joseph’s University Medical Center in Patterson, Henry J. Austin Health Center at Capital Health in Trenton, Waymon C. Lattimore Practice at Rutgers Global Tuberculosis Institute in Newark, and Rutgers Health in New Brunswick and ID Care, with medical office locations throughout New Jersey including Princeton, Somerset, Hillsborough, East Brunswick, Oakhurst, Wayne, Old Bridge, Cedar Knolls, and Randolph. Furthermore, community outreach efforts extended to Newark and Irvington senior centers. Community-based non-profit organizations involved in distributing the survey to broader New Jersey community members included the South Asian Total Health Initiative (SATHI) at Rutgers Robert Wood Johnson Medical School, focusing on the South Asian community throughout New Jersey. We included people over the age of 18 who identified themselves as Asians (Indian, Pakistani, Chinese, Korean, Bangladeshi, Filipino, Bhutanese, Vietnamese, Japanese, and other Asians) throughout the state (*N* = 157). Participants were excluded if essential data elements such as vaccine behaviors, reluctance, trust, and socioeconomic status were missing or incomplete in their survey records.

### Measurements

A review was conducted and the predictor variables representing SES were identified as education, household income and employment [5, 9, 10]. Education level was categorized as Less than 12th grade, High School (completed 12th grade), some college, Associates, Bachelors, Masters, and Doctorate. Household income comprises of nine categories i.e., Unsure, less than $20,000, $21–39,000, $40–59,000, $60–79,000, $80–99,000, $100–149,000, $150,000 and more, and Prefer not to say. Additionally, Employment status has two set of responses i.e., Yes and No. Employment type has six categories namely Full-time, Part-time, Homemaker, Retired/disabled, Student and Unemployed.

### Outcomes

Our primary aim is to evaluate whether SES is associated with vaccination behaviors/attitudes. The outcome variables consist of adult vaccines measures (influenza, pneumococcal and COVID-19) including vaccine attitudes. Vaccine attitudes has two categories namely Yes and No wherein participants were asked whether they were reluctant or hesitant to receive any vaccine. Vaccine confidence and trust have ordinal scale rating as A lot, A little or some, none at all, not very much and do not know [5, 9, 10].

### Data Analysis

Descriptive and inferential statistics were performed. Median and interquartile range were reported to analyze continuous variables such as age based on normality of distribution, whereas categorical variables including race, sex, education, household income, employment status and type were summarized using frequencies and percentages. Fisher’s Exact test or Chi square test was used to analyze and stratify outcome variable (vaccine hesitancy) with SES variables including education level, household income, employment status and type. Multivariable logistic regression was used to analyze the association between vaccine hesitancy and various predictor variables. Confounding was assessed using the backward selection process and theoretical plausibility. Moreover, influenza, pneumococcal, and COVID-19 vaccine confidence ordinal scale frequency were analyzed. Statistical results were reported in the form of tables along with their *p*-values. As standard, *p*-values > 0.05 will not be considered statistically significant. Data analysis was conducted using SAS software version 9.4 (SAS Institute, Cary, NC, USA).

## Results

In this study, we analyzed 157 individuals for our analytic sample who identified themselves belonging to Asian race having age ≥ 18 years. Table 1 provides baseline characteristics of the sample population. The median age of the participants was 24 years with an interquartile range (IQR) of 22–30 years. Among the participants, 62.42% were male and 37.58% were female. Most participants (28.70%) were of Indian origin, followed by Chinese (12.73%), Korean (10.20%), Pakistani (7.64%), Filipino (5.10%), Bangladeshi (3.20%), Sri Lankan (2.00%), Vietnamese (1.30%), and other Asian origins (29.30%). The median household number was 4 with an IQR of 3–4. 54.78% were born in the US. 97.45% had received at least one COVID-19 vaccine dose, with a median of 3 doses (IQR 3–3). The most common COVID-19 vaccine types were Pfizer (59.5%) and Moderna (36%), while only 4.58% received the J&J/Janssen vaccine. The majority of participants (79%) received their vaccines in 2021 [Table 1].

**Table 1:** Baseline characteristics

Baseline Characteristics	Total analytic sample (*N* = 157)
Age (years)*	24 (22–30)
Gender**	
Males	98 (62.42)
Females	59 (37.58)
Asian Race Subcategories**	
Indian	45 (28.70)
Chinese	20 (12.73)
Korean	16 (10.20)
Pakistani	12 (7.64)
Filipino	8 (5.10)
Bangladeshi	5 (3.20)
Sri Lankan	3 (2.00)
Vietnamese	2 (1.30)
Other Asians	46 (29.30)
Household Number*	4 (3–4)
Birthplace**	
US born	86 (54.78)
Not Born in the US	68 (43.31)
Prefer not to say	3 (1.91)
Total COVID-19 Vaccine doses (Including Boosters)*	3 (3–3)
COVID-19 Vaccine Status**	
Yes	153 (97.45)
No	4 (2.55)
COVID-19 Vaccine Type**	
J&J/Janssen	7 (4.58)
Moderna	55 (36.00)
Pfizer	91 (59.50)
COVID-19 Vaccine Year**	
2020	31 (21.00)
2021	117 (79.00)

*Continuous variables reported as median and Interquartile range (IQR)

**Categorical variables reported as frequency n (%)

Among the total sample, 19 individuals (12.1%) reported being vaccine hesitant or reluctant, while the remaining 138 individuals (87.9%) reported no vaccine hesitancy. There was no statistically significant association between vaccine hesitancy and Education (*p* = 0.68). Participant’s educational level revealed that the majority had completed bachelor’s degree (30.62%), followed by those with some college education (23.0%), completing high school (15.9%), Doctorate degree (14.6%), while a smaller proportion reported having an associate degree (6.4%), a master’s degree (8.9%), with (0.6%) reporting less than a 12th grade education. With regards to employment status, 125 (79.6%) reported being employed with no statistically significant association (*p* = 1.0). When considering employment type, most participants (38.2%) were employed full-time, followed by students (32.5%) and those employed part-time (23.6%). A smaller number of participants (2.0%) reported being retired or disabled, unemployed (3.2%), or a homemaker (0.6%), with no statistically significant association with vaccine hesitancy and employment type (*p* = 0.48). Regarding household income, 21.66% reported having an income of $150,000 or more, 15.92% having an income of $60,000-$79,000, and 13.4% belonging to $100,000-$149,000 range, with 9.5% reporting less than $20,000, 8.9% reporting an income range of $40,000-$59,000, 7.0% reporting an income range of $80,000-$99,000, and 5.7% reporting an income range of $21,000-$39,000. Furthermore, 7.6% preferred not to reveal their income, and 10.2% were unsure of their income range. However, no statistically significant difference was observed between household income and vaccine hesitancy (*p* = 0.15). Multivariable logistic regression modeling confirmed that none of the SES predictor variables were associated with vaccine hesitancy [Table 2]. However, as an exploratory finding, gender was found to be a significant predictor while adjusting for age, birthplace, and religion with males having lower odds of vaccine hesitancy than females (adjusted OR = 0.14; *p* < 0.05).

**Table 2:** Bivariate analysis of SES stratified by vaccine hesitancy

Socio-Economic Status (SES)	Total Analytic Sample (*N* = 157)	Vaccine Hesitant/ Reluctant (*N* = 19)	No Vaccine Hesitancy or Reluctancy (*N* = 138)	*P*-value
Education*				0.68
Associates	10 (6.40)	1 (5.26)	9 (6.52)	
Bachelors	48 (30.60)	7 (36.84)	41 (29.71)	
Masters	14 (8.92)	3 (15.8)	11 (7.97)	
Some College	36 (23)	5 (26.32)	31 (22.46)	
High School (Completed 12th Grade)	25 (15.9)	1 (5.26)	24 (17.4)	
Less than 12th Grade	1 (0.64)	0 (0)	1 (0.72)	
Doctorate	23 (14.65)	1 (14.30)	22 (14.67)	
Employment Status*				1.0
Yes	125 (79.62)	15 (78.95)	110 (79.71)	
No	32 (20.40)	4 (21.00)	28 (20.30)	
Employment Type*				0.48
Full-time employment	60 (38.22)	8 (42.11)	52 (37.68)	
Homemaker	1 (0.64)	0 (0)	1 (0.72)	
Part-time employment	37 (23.60)	6 (31.60)	31 (22.46)	
Retired/disabled	3 (2)	1 (5.26)	2 (1.45)	
Student	51 (32.50)	4 (21)	47 (34)	
Unemployed	5 (3.18)	0 (0)	5 (3.62)	
Household Income*				
Less than $20,000	15 (9.50)	0 (0)	15 (10.87)	0.15
$21,000-$39,000	9 (5.73)	1 (5.26)	8 (5.8)	
$40,000-$59,000	14 (8.90)	2 (10.53)	12 (8.7)	
$60,000-$79,000	25 (15.92)	4 (21)	21 (15.22)	
$80,000-$99,000	11 (7)	1 (5.26)	20 (14.5)	
$100,000-$149,000	21 (13.40)	1 (5.26)	32 (23.2)	
$150,000 and more	34 (21.66)	2 (20.53)	32 (23.2)	
Prefer not to say	12 (7.64)	4 (21)	8 (5.8)	
Unsure	16 (10.20)	4 (21)	12 (8.7)	

*Categorical variables reported as n (%)

Table 3 presents the findings of the study assessing the level of confidence in taking influenza, pneumococcal, before and during the COVID-19 pandemic along with COVID-19 vaccine confidence. The findings indicate that a significant proportion of participants had confidence in taking these vaccines before the pandemic, with confidence levels increasing during the pandemic. Prior to the pandemic, 62.3% and 59.2% of participants expressed high confidence in taking the influenza and pneumococcal vaccines, respectively. During the pandemic, these percentages increased to 70.1% and 70.5%, respectively. The majority of respondents reported a high level of confidence in taking the COVID-19 vaccine, with 114 individuals (73.1%) indicating they had ‘’a lot of confidence’’. A smaller number of respondents, 27 (17.3%), reported having ‘’a little or some level of confidence’’, while 8 (4.5%) were uncertain and 7 (4.5%) reported having ‘’not very much’’ confidence. Only one individual (0.6%) reported having ‘’none at all’’ confidence in taking the COVID-19 vaccine currently.

**Table 3:** Vaccine (influenza, pneumococcal, and COVID-19) confidence gaps summary

Vaccine confidence gaps summary*					
*Confidence Scale*	*How much confidence do you have in taking the* ***Flu*** *vaccine?*		*How much confidence do you have in taking the* ***Pneumococcal*** *vaccine?*		*How much confidence do you have in taking the* ***COVID-19*** *vaccine currently?*
	Before COVID-19	After COVID-19	Before COVID-19	After COVID-19	During/After COVID-19
A lot	96 (62.34)	108 (70.13)	93 (59.20)	110 (70.51)	114 (73.10)
A little or some	35 (22.73)	24 (25.6)	26 (16.56)	25 (16.03)	27 (17.31)
Do not Know	10 (5)	8 (5.2)	23 (14.65)	12 (7.70)	8 (4.50)
Not very much	12 (7.8)	13 (8.44)	8 (5.10)	9 (5.77)	7 (4.45)
None at all	4 (2.6)	1 (0.65)	7 (4.46)	0 (0)	1 (0.64)

*Categorical variables reported as n (%)

## Discussion

The study analyzed individuals of Asian origin aged 18 years or older to determine their vaccine related behaviors and attitudes. Most of the participants had received at least one dose of the COVID-19 vaccine, with no significant association observed between vaccine hesitancy and SES. The study also found that reported confidence levels in taking influenza and pneumococcal vaccines increased during the pandemic. These findings suggest that addressing vaccine hesitancy should be targeted towards all individuals regardless of their SES for Asian Americans reflective of our sample.

The findings suggests that most Asian individuals in the study are vaccinated to protect themselves and other from COVID-19 and other infectious diseases. The high confidence for various vaccines captures that the pandemic has heightened awareness of the importance of vaccination and may have helped increase public confidence in vaccines overall. The fact that most participants in the study received at least one dose of the COVID-19 vaccine with a median of 3 doses (IQR 3–3) signifies the awareness of booster doses in the Asian community showing active engagement in vaccination efforts. This is an important finding as vaccination is one of the key strategies to control the spread of COVID-19 and other infectious diseases. Our study’s findings are supported in prior research by Malik, Amyn A., et al. which mentions that majority of the population (67%) in the US would accept COVID-19 vaccine [11]. 

One potential limitation of this study is its sample size and age distribution, which may limit its generalizability to other populations within NJ as the results may not accurately reflect the diversity and heterogeneity within the Asian-American population. Additionally, the education level in our sample was high and likely differ from that of the general NJ population. The results of a convenience sample within New Jersey may not be representative of the larger Asian-American population in other regions or states. Another potential limitation is its reliance on self-reported data, which may introduce recall bias or inaccuracies. Future studies should aim to address these limitations by using larger Asian-American cohorts, objective measures (e.g., electronic medical record data), and mixed methods designs to gather more comprehensive data.

Despite its limitations, this study’s strengths include its focus on a specific population and its use of standardized measures to assess vaccine hesitancy. Furthermore, this study addresses the research gap in the literature as data are limited for Asian-Americans and their health behaviors. These findings have important public health implications, particularly for policymakers and healthcare providers working to increase vaccine uptake among underserved populations. Future studies should aim to build upon these findings by exploring additional factors that may contribute to vaccine hesitancy and developing targeted interventions to address these concerns.

## Declarations

### Previous Presentations

Presented in part as a poster presentation at American Public Health Association (APHA) conference in November 2023, Atlanta, Georgia, USA.

## References

[1] Tur-Sinai, A., Gur-Arie, R., Davidovitch, N., Kopel, E., Glazer, Y., Anis, E., & Grotto, I. (2019). Vaccination uptake and income inequalities within a mass vaccination campaign. *Israel Journal of Health Policy Research*, *8*(1), 63–63. 10.1186/s13584-019-0324-6 31307532 10.1186/s13584-019-0324-6 PMC6628472

[2] Fridman, A., Gershon, R., & Gneezy, A. (2021). COVID-19 and vaccine hesitancy: A longitudinal study. *PloS One*, *16*(4), e0250123–e0250123. 10.1371/journal.pone.0250123 33861765 10.1371/journal.pone.0250123 PMC8051771

[3] Bertoncello, C., Ferro, A., Fonzo, M., Zanovello, S., Napoletano, G., Russo, F., Baldo, V., & Cocchio, S. (2020). Socioeconomic determinants in Vaccine Hesitancy and Vaccine Refusal in Italy. *Vaccines (Basel)*, *8*(2), 276. 10.3390/vaccines8020276 32516936 10.3390/vaccines8020276 PMC7349972

[4] Saban, M., Myers, V., Ben-Shetrit, S., & Wilf-Miron, R. (2021). Socioeconomic gradient in COVID-19 vaccination: Evidence from Israel. *International Journal for Equity in Health*, *20*(1), 242. 10.1186/s12939-021-01566-4 34749718 10.1186/s12939-021-01566-4 PMC8574141

[5] Larson, H. J., de Figueiredo, A., Xiahong, Z., Schulz, W. S., Verger, P., Johnston, I. G., Cook, A. R., & Jones, N. S. (2016). The state of Vaccine confidence 2016: Global insights through a 67-Country survey. *EBioMedicine*, *12*, 295–301. 10.1016/j.ebiom.2016.08.042 27658738 10.1016/j.ebiom.2016.08.042 PMC5078590

[6] Pastorino, R., Villani, L., La Milia, D. I., Ieraci, R., Chini, F., Volpe, E., Barca, A., Fusco, D., Laurenti, P., Ricciardi, W., & Boccia, S. (2021). Influenza and pneumococcal vaccinations are not associated to COVID-19 outcomes among patients admitted to a university hospital. *Vaccine*, *39*(26), 3493–3497. 10.1016/j.vaccine.2021.05.015 34020813 10.1016/j.vaccine.2021.05.015 PMC8106908

[7] Daniels, N. A., Gildengorin, G., Nguyen, T. T., Liao, Y., Luong, T. N., & McPhee, S. J. (2010). Influenza and pneumococcal vaccination rates among Vietnamese, Asian, and non-hispanic white americans. *Journal of Immigrant and Minority Health*, *12*(3), 370–376. 10.1007/s10903-008-9195-6 18839311 10.1007/s10903-008-9195-6 PMC4075262

[8] Hrywna, M., Jane Lewis, M., Mukherjea, A., Banerjee, S. C., Steinberg, M. B., & Delnevo, C. D. (2016). Awareness and use of south Asian Tobacco products among South asians in New Jersey. *Journal of Community Health*, *41*(6), 1122–1129. 10.1007/s10900-016-0208-4 27256410 10.1007/s10900-016-0208-4 PMC5083141

[9] Larson, H. J., Schulz, W. S., Tucker, J. D., & Smith, D. M. (2015). Measuring vaccine confidence: introducing a global vaccine confidence index. *PLoS currents*, *7*, ecurrents.outbreaks.ce0f6177bc97332602a8e3fe7d7f7cc4. 10.1371/currents.outbreaks.ce0f6177bc97332602a8e3fe7d7f7cc4 10.1371/currents.outbreaks.ce0f6177bc97332602a8e3fe7d7f7cc4 PMC4353663 25789200

[10] Larson, H. J., Jarrett, C., Schulz, W. S., Chaudhuri, M., Zhou, Y., Dube, E., Schuster, M., MacDonald, N. E., Wilson, R., & SAGE Working Group on Vaccine Hesitancy. (2015). Measuring vaccine hesitancy: The development of a survey tool. *Vaccine*, *33*(34), 4165–4175. 10.1016/j.vaccine.2015.04.037 25896384 10.1016/j.vaccine.2015.04.037

[11] Malik, A. A., McFadden, S. M., Elharake, J., & Omer, S. B. (2020). Determinants of COVID-19 vaccine acceptance in the US. *EClinicalMedicine*, *26*, 100495. 10.1016/j.eclinm.2020.100495 32838242 10.1016/j.eclinm.2020.100495 PMC7423333

